# From Growth Mindsets to Life Satisfaction: Examining the Role of Cognitive Reappraisal and Stressful Life Events

**DOI:** 10.3390/healthcare13222985

**Published:** 2025-11-20

**Authors:** Rahma F. Goran, Xu Jiang

**Affiliations:** Department of Psychological Studies in Education, College of Education and Human development, Temple University, 1301 Cecil B Moore Ave., Philadelphia, PA 19122, USA; rahma.goran@temple.edu

**Keywords:** growth mindset, stress, emotion regulation, cognitive reappraisal, life satisfaction

## Abstract

**Background/Objectives:** Implicit theories of thoughts, emotions, and behavior (TEB) describe beliefs that these attributes are either changeable (growth mindset) or unchangeable (fixed mindset). While the impact of mindsets on negative mental health indicators, such as psychopathological symptoms, is well-documented, their relations with positive indicators such as life satisfaction, particularly in the context of stress, remain underexplored. This study aimed to address this gap by testing whether the association between adolescents’ implicit theories of TEB and life satisfaction is mediated by cognitive reappraisal and whether stressful life events moderated two paths within the mediation model. **Methods:** Participants were 620 high school students (49.5% female, 43.5% male, 5.8% gender-nonconforming, 1.1% undisclosed) aged 14 to 19 years (M = 17.51, SD = 1.23), who completed an online survey in Spring 2022, while the COVID-19 pandemic still significantly affected daily life. Mediation and moderated mediation models were tested using PROCESS macro in SPSS. **Results:** Mediation analysis revealed that growth mindset positively influenced life satisfaction both directly and indirectly through cognitive reappraisal. Stressful life events significantly moderated the direct effect of growth mindset on life satisfaction, with the positive direct effect diminishing as stress increased. **Conclusions:** The positive link between growth mindset and life satisfaction was strongest under lower stress and transmitted through cognitive reappraisal across stress levels. Given the cross-sectional design, findings should be interpreted as correlational, not causal. Future longitudinal research should clarify temporal directionality and reciprocal links among mindset, coping, and well-being to inform interventions that strengthen adaptive beliefs and regulation skills.

## 1. Introduction

Adolescence is a time of major change—biologically, psychologically, and socially. As teens gain the ability to think more abstractly and reflect on their thoughts, emotions, and behavior, they also face mounting demands across school, family, and peer contexts. These challenges make adolescence a particularly vulnerable period for stress and the emergence of mental health difficulties; many internalizing and externalizing disorders first appear during this stage [1]. Contemporary developmental work characterizes adolescence as a sensitive window for the onset of “social–emotional” disorders such as social anxiety, depression, generalized anxiety, and eating disorders, with marked increases in these problems from early to mid-adolescence, especially among girls [2]. Population-based studies similarly show that a sizable minority of adolescents report significant psychological difficulties, with recent evidence indicating that approximately 15–20% experience clinically meaningful emotional or behavioral symptoms during this period [3]. Understanding what belief systems help teens make sense of stress and regulate themselves effectively is therefore a central developmental question.

The COVID-19 pandemic provided an unprecedented real-world context for exploring this question. School closures, remote learning, disrupted routines, and heightened family stress collectively strained adolescents’ adaptive systems, producing a sustained period of uncertainty and emotional challenge. Rather than functioning solely as background context, the pandemic represents a large-scale naturalistic test of how belief and coping processes operate under chronic adversity. From a resilience perspective, COVID-19 disrupted multiple ecological systems that typically support well-being, i.e., family, school, and community, thereby revealing individual differences in how youth mobilize internal resources such as coping flexibility and emotion regulation [4]. This natural experiment underscores the importance of examining how adolescents’ beliefs about personal change shape their ability to adapt when external supports are constrained.

Within stress-appraisal theory, these belief systems play a central role. How individuals interpret stressors and evaluate their capacity to manage them determines whether stress responses become adaptive or maladaptive [5,6]. Developmental research further shows that the capacity for flexible appraisal and regulation is scaffolded by supportive environments that foster coping confidence and self-efficacy [7,8]. When those external systems are taxed—as during the pandemic—beliefs about control and change may become particularly salient in guiding how adolescents construe stress and select coping strategies.

Mindset theory provides a framework for understanding these beliefs. It describes the implicit beliefs people hold about whether attributes are fixed (entity beliefs) or changeable (incremental beliefs) [9,10]. Mindset theory was first applied to intelligence, where studies showed that adolescents who believed intelligence could grow with effort achieved higher grades and handled setbacks more constructively [11]. Later, meta-analytic evidence demonstrated that growth mindsets are consistently linked to the self-regulation processes that support success, like setting challenging goals, persisting after setbacks, and sustaining effort when tasks are difficult [12]. Extensions into social and emotional domains have found similar effects: adolescents who believe personality is malleable are more likely to handle social stress in healthier ways [13]. Reviews of the broader literature confirm that growth mindset is associated with better academic and mental health outcomes in adolescents and can be shifted through intervention [14,15].

Importantly, scholars now distinguish between *domain-general* and *domain-specific* mindsets. Domain-general mindsets refer to overarching beliefs about whether human attributes, in general, can change, whereas domain-specific mindsets apply to particular areas of functioning such as intelligence, emotion, or behavior [12,13]. This distinction matters because the predictive power of mindset beliefs depends on how well the belief domain aligns with the outcome of interest. For instance, while general growth beliefs may foster broad resilience, beliefs about the malleability of emotions are more directly tied to emotional regulation and well-being [16,17,18,19]. The present study focuses on mental health mindsets—beliefs about the changeability of one’s own thoughts, emotions, and behaviors—as a domain-specific construct that more precisely captures how adolescents interpret and respond to internal challenges.

Early work on implicit theories of emotion showed that beliefs about emotion predict important outcomes in young people. For instance, first-year college students who endorsed more fixed beliefs about emotion reported lower self-efficacy in emotion regulation, more negative experiences, higher depressive symptoms, and lower well-being at the end of the year [16]. Similarly, undergraduates with more fixed emotion beliefs reported lower reappraisal use, lower self-esteem and life satisfaction, and higher depression and stress [17]. Studies that included adolescents and young adults found that more growth-oriented beliefs about emotions were associated with greater use of cognitive appraisal strategies [18,19], more positive affect [20] and higher self-perceived emotional competence [21]. Together, these findings suggest that implicit theories about emotions shape how youth regulate and experience emotions, with consequences for mental health.

Building on this, Schleider and Weisz [22,23] introduced the implicit theories of thoughts, emotions, and behavior (ITEB) framework, which applies the mindset concept to domains central to mental health. In an early adolescent sample (ages 11–14), they developed the Implicit Theories of Thoughts, Emotions, and Behavior Questionnaire (ITEB-Q), finding acceptable reliability and evidence that stronger fixed beliefs predicted increases in internalizing symptoms over time [22]. More recently, psychometric work in a large, diverse U.S. high-school sample corroborated the ITEB-Q’s three-factor structure, established scalar invariance across gender and Black/White groups, and demonstrated concurrent validity with life satisfaction, cognitive reappraisal, resilience, and emotional problems [24]. In a separate U.S. high-school study, the association between family stress and externalizing was attenuated for youth endorsing stronger growth-oriented ITEB beliefs [25].

The inclusion of life satisfaction as a primary positive outcome reflects both theoretical and empirical considerations. From a stress-appraisal perspective, life satisfaction captures adolescents’ global evaluation of their circumstances relative to their goals and resources [5,26]. It integrates cognitive and emotional appraisals of well-being, making it a sensitive indicator of successful adaptation under stress. Prior studies using the ITEB framework have consistently shown that growth-oriented beliefs are linked to higher life satisfaction even when accounting for emotional problems [24], suggesting that mindset-related differences extend beyond the reduction in distress to the presence of positive psychological functioning. Thus, life satisfaction offers a meaningful and theoretically coherent indicator of adolescents’ adaptive adjustment within a mindset-coping framework.

Collectively, these findings position ITEB as a developmentally relevant and clinically actionable framework for understanding how adolescents construe internal challenges, especially under conditions of heightened stress like the pandemic.

### 1.1. A Framework for Linking Beliefs, Coping, and Well-Being

Research increasingly shows that ITEB beliefs shape how adolescents interpret stress and regulate emotions, with meaningful implications for mental health. These beliefs are not fixed traits: longitudinal evidence indicates that increases in internalizing symptoms predict stronger fixed beliefs over time, suggesting a reciprocal risk process [22]. At the same time, brief single-session interventions designed to promote growth beliefs can shift these beliefs and reduce depressive symptoms [23]. Taken together, these findings position ITEB as both a marker of vulnerability and a modifiable prevention target during a developmental window when belief systems and regulatory skills are still consolidating.

The Setting-Operating-Monitoring-Achievement (SOMA) model provides a useful framework for organizing how such beliefs steer self-regulation [12,26]. In SOMA, goal setting involves adopting learning versus performance aims, goal operating captures the strategies used to pursue those aims, and goal monitoring refers to how progress is evaluated. Across more than 28,000 participants, meta-analytic evidence shows that incremental mindsets predict mastery-oriented goals, persistence in the face of setbacks, and optimistic evaluations of progress, with effects strongest under stress [12]. Although SOMA was developed largely in academic contexts, its principles generalize across domains, making it a strong candidate for conceptualizing ITEB in adolescence.

When applied to mental health, SOMA suggests that ITEB functions as a motivational lens that shapes stress appraisals (setting), channels coping strategies that enact beliefs (operating), and drives outcomes like depressive symptoms and life satisfaction (achievement). Among these mechanisms, cognitive reappraisal represents a particularly proximal and theoretically coherent pathway. Adolescents who endorse malleable beliefs about emotions report greater use of reappraisal, which in turn predicts lower depressive symptoms in both cross-sectional and longitudinal studies [27,28]. Reappraisal also fits contemporary accounts of emotion-regulation flexibility—the capacity to deploy context-appropriate strategies—by allowing youth to reinterpret stressors rather than avoid them [29]. Experimental work demonstrates that inducing incremental emotion beliefs shifts state regulation toward greater use of adaptive strategies such as reappraisal [18]. Beyond its frequency of use, reappraisal reflects emotion-regulation flexibility—the ability to adapt strategies to shifting emotional demands [29]. Recent evidence demonstrates that positive cognitive reappraisal flexibility, or the adaptive adjustment of reappraisal tactics to meet situational emotional demands, predicts reductions in perceived stress over time [30], strengthening the argument that reappraisal mediates mindset–well-being links not only because it reduces negative affect but also because it enables flexible, context-attuned regulation.

Converging findings across mindset and stress-regulation research further highlight the domain-specific nature of these mechanisms. In stress-mindset research, endorsing a “stress-is-enhancing” mindset has been shown to foster approach-oriented physiological and affective responses through reappraisal processes [31]. In the academic domain, growth mindset about learning ability predicts greater cognitive reappraisal and, in turn, higher vitality, academic buoyancy, and life satisfaction [32]. Likewise, in a study of undergraduates, a growth mindset about emotion and self-regulation amplified the benefits of reappraisal for mental wellness [33]. Across domains, these findings underscore that the belief in change—whether applied to stress, learning, or emotion—facilitates flexible reinterpretation of stressors and supports positive affective functioning. Collectively, this evidence positions cognitive reappraisal as a mechanism that translates malleability beliefs into not only lower distress but also sustained well-being and positive emotional engagement.

This mechanism also aligns with the transactional model of stress and coping, which frames appraisal as the cognitive bridge between stress exposure and adaptive responses [34]. From this perspective, mindset beliefs may influence primary appraisals (e.g., whether stressors are viewed as controllable or threatening), whereas reappraisal captures secondary, flexible reinterpretations that reshape emotional outcomes. In contrast, fixed emotion beliefs are linked to reduced reappraisal, greater reliance on suppression, and heightened depressive symptoms [27]. Converging evidence further indicates that beliefs in emotional controllability predict more reappraisal and less suppression, which is associated with lower anxiety and depression [35], and that positive cognitive reappraisal is reliably related to stress resilience, mental health, and positive affect [36]. Thus, cognitive reappraisal represents a theoretically central operating mechanism through which ITEB exerts its effects on both emotional adaptation and flourishing.

Contextual conditions influence these pathways. Consistent with SOMA’s prediction that mindset effects are strongest under challenge, studies show that supportive environments allow adolescents to enact adaptive beliefs, while high-stress contexts can constrain these processes [16]. Incremental beliefs of mental health, for example, have been shown to buffer the link between family stress and externalizing problems in American adolescents [25]. Another U.S. study likewise found that growth beliefs correlate positively with resilience, reappraisal, and life satisfaction, and negatively with emotional problems [24]. Notably, associations with well-being indicators were at least as strong as those with symptoms, underscoring ITEB’s role in promoting positive outcomes as well as reducing risk. Still, naturalistic work during the COVID-19 pandemic suggests that even adaptive strategies like reappraisal may be insufficient to fully offset distress under pervasive, chronic stress, whereas maladaptive strategies can exacerbate difficulties [37]. Stress, then, acts as a contextual moderator that can amplify or dampen the influence of ITEB on adolescent adjustment.

Finally, it is important to view adolescent mental health through a dual-factor lens. Mental health cannot be reduced to the absence of distress; well-being and symptoms represent related but distinct dimensions [38]. Life satisfaction—youth’s global appraisal of their family, peer, school, and self domains [39]—is a particularly important indicator. Motivational strengths such as grit have been linked to higher life satisfaction [40], and evidence suggests that growth-oriented mental health or emotion beliefs predict not only fewer internalizing and externalizing problems but also higher life satisfaction [21,24,26]. In this study, we therefore prioritize cognitive reappraisal among candidate mediators because it (a) is malleable and demonstrably influenced by mindset inductions [18], (b) reflects emotion-regulation flexibility, a core component of effective stress adaptation [29], and (c) shows robust links to positive affect and well-being, including life satisfaction [27,35,36]. This dual role highlights ITEB as a framework that captures both risk and resilience, offering insight into how beliefs about personal change can guide coping, buffer stress, and ultimately foster flourishing in adolescence.

### 1.2. Current Study

The current study aims to clarify how beliefs, coping strategies, and contextual stressors intersect in shaping adolescent subjective well-being. Specifically, we examine how mental-health growth mindsets (ITEB)—beliefs about the malleability of one’s thoughts, emotions, and behaviors—relate to life satisfaction through the mechanism of cognitive reappraisal, and whether these associations vary by perceived stress. Although the COVID-19 pandemic is not the focus of the present analyses, it provides an important contextual backdrop that heightened stress exposure and challenged regulatory systems, thereby underscoring the need to understand belief–coping processes under sustained adversity [4]. By employing a moderated mediation model, we examine whether adolescents with stronger ITEB growth beliefs report higher life satisfaction, whether cognitive reappraisal explains this association, and whether stress alters the strength of these links. This approach allows us to examine both direct and indirect pathways between beliefs and well-being, while also situating these processes within stress-appraisal theory and ecological models of resilience.

We extend prior research in three ways. First, we use life satisfaction as a positive indicator of well-being rather than traditional indicators which are deficit-oriented (e.g., distress). Second, guided by self-regulatory theory and the evidence above, we focus on cognitive reappraisal as the mediating process because it is theoretically proximal to emotion-related mindsets, experimentally responsive to mindset interventions, and conceptually linked to emotion-regulation flexibility and positive affect [18,29,30,31,32,33]. Third, we examine perceived stress as a contextual moderator to assess whether the benefits of adaptive beliefs and reappraisal vary by stress level—enhancing well-being under manageable stress but diminishing under chronic, high-stress conditions.

Based on these objectives, we hypothesize that:

(**H1**)  *Adolescents endorsing stronger ITEB growth beliefs will report higher life satisfaction.*

(**H2**)  *This association will be mediated by cognitive reappraisal, such that growth-oriented beliefs predict greater reappraisal use, which in turn predicts higher life satisfaction.*

(**H3**)  *Perceived stress will moderate this indirect pathway, such that the positive effect of growth beliefs on reappraisal and well-being will be attenuated at higher stress levels.*

Together, these objectives and hypotheses position the study to test an integrated model of belief, coping, and context that advances understanding of adolescent resilience during a period of heightened environmental stress. The findings help lay groundwork for future longitudinal and intervention research.

## 2. Materials and Methods

### 2.1. Participants and Procedure

The present study used a pre-existing, cross-sectional dataset, from a broader research initiative/study that examines stress-related growth in adolescents and young adults amid the COVID-19 pandemic. The present study aimed to capture a snapshot of high school students’ experiences during their transition from in-person to virtual instruction amid the COVID-19 pandemic. Approval for this larger project was obtained from the Institutional Review Board at the Temple University. Data collection occurred through the utilization of the Qualtrics research panel, a well-regarded online recruitment platform. The survey administration took place after students had resumed in-person schooling post-COVID (Spring 2022), and participants were tasked with completing an online self-report questionnaire.

The study participants comprised high school students from the United States who had undergone the transition from in-person to virtual instruction for a minimum of six months during the COVID-19 pandemic. From the initial pool of 630 respondents, ten individuals were excluded in the subsequent data analysis. Exclusion criteria involved the removal of eight respondents due to duplicate IP addresses and the exclusion of two respondents who had completed the survey in under six minutes. Thus, the final sample consisted of 620 high school students enrolled in grades 9th through 12th. Their ages ranged from 14 to 19 years old (M = 17.51, SD = 1.23). The study included students from grades 9 to 12, with a diverse representation across gender, race, and geographic regions. The majority of participants attended suburban and urban schools, with a smaller percentage from rural areas. The sample was nearly evenly split by gender, with a small percentage identifying as gender-nonconforming or choosing not to disclose their gender. Most students lived with both parents, with others living in various family structures. Racially, the sample included students from a range of backgrounds, including White, Black, Hispanic/Latinx, Asian, and other racial identities. Geographically, participants came from the South, West, Midwest, and Northeast regions of the United States (see Table 1).

Importantly, the pandemic context served not merely as background but as a naturalistic stress test for examining belief–coping interactions under sustained adversity [4]. This period disrupted multiple ecological systems that typically support adolescent adjustment—family, school, and community—thereby amplifying the relevance of internal belief systems such as mental health growth mindsets and coping flexibility as adaptive resources. Consistent with the SOMA model [12] and stress-appraisal theory [5], the present study conceptualizes mindset as an upstream cognitive lens that guides stress appraisal and coping behaviors, which subsequently influence well-being outcomes. Prior research supports this directional order, showing that incremental emotion or mental health mindsets predict greater cognitive reappraisal and higher life satisfaction in adolescents and young adults [15,18,28,35].

Given that no values were missing on the key study variables, no imputation or missing-data adjustments were required. Analyses were conducted using Hayes’ [41] PROCESS macro (Model 14) in IBM SPSS Statistics (Version 29), with 5000 bootstrap samples and 95% bias-corrected confidence intervals. No control variables were included, as preliminary analyses indicated that demographic variables (e.g., gender, age, race) were not significantly correlated with the primary variables of interest.

All relevant assumptions for multiple regression were evaluated and met. Scatterplots and residual plots supported linearity and homoscedasticity; residuals were approximately normally distributed. Variance inflation factors (all VIFs ≈ 1.00; Tolerance ≥ 0.99) indicated no evidence of multicollinearity among ITEB, perceived stress, or their interaction term. Because the PROCESS macro employs bootstrapping, normality of the indirect effect distribution was not assumed [41].

### 2.2. Measures

#### 2.2.1. Implicit Theories of Thoughts, Emotion, and Behavior

The Implicit Thoughts, Emotion, and Behavior Questionnaire (ITEB-Q) [22] draws inspiration from Dweck’s research on implicit theories of personality [42,43]. Comprising twelve items, the scale is distributed across three categories: thoughts, emotions, and behaviors, each containing four items. All items within each subgroup encapsulate beliefs aligned with an extreme growth mindset. For instance, a representative item reads, “You can change how you behave if you really try.” Using a four-point Likert scale, youths express their level of agreement with each statement, ranging from 1 (Very False) to 4 (Very True). Subscale sum scores were computed and averaged to gauge the intensity of beliefs pertaining to the adaptability of one’s thoughts, emotions, and behaviors. The scale’s reliability and validity were initially established through recent studies [22,23], affirming its psychometric soundness. The internal consistency of the ITEB-Q was high, with Cronbach’s alpha coefficient being α = 0.89 and McDonald’s omega coefficient being ω = 0.89.

#### 2.2.2. Cognitive Reappraisal

The Emotion Regulation Questionnaire for Children and Adolescents (ERQ-CA) [44] represents a refined adaptation of the ERQ [45], originally formulated to explore emotion regulation strategies in adults. Noteworthy revisions were implemented in the ERQ-CA, including the simplification of item phrasing (e.g., altering “I control my emotions by not expressing them” to “I control my feelings by not showing them”), alongside the truncation of the response scale to encompass five points (ranging from 1 = strongly disagree to 5 = strongly agree). Consisting of a total of 10 items, the ERQ-CA is tailored to assess two distinct emotion regulation strategies: cognitive reappraisal, comprising 6 items, and expressive suppression, encompassing 4 items. Elevated scores on each subscale denote a heightened application of the respective emotion regulation strategy. The ERQ-CA showcases robust psychometric characteristics, as evidenced by its internal consistency, stability over a 12-month span, and its construct and convergent validity, as established through research endeavors [44,46]. The cognitive reappraisal subscale demonstrated satisfactory internal consistency, with Cronbach’s alpha coefficient of α = 0.78 and McDonald’s omega coefficient of ω = 0.77.

#### 2.2.3. Life Satisfaction

The Brief Multidimensional Students’ Life Satisfaction Scale (BMSLSS) [47] is a concise self-assessment tool designed to gauge the contentment of children and adolescents across dimensions relevant to their developmental stage. This measurement consists of five items, prompting students to evaluate their satisfaction levels in key areas of youth life, including family, friendships, school experiences, self-perception, and living environment. Participants rated their satisfaction on a 7-point rating scale ranging from 1 (terrible) to 7 (delighted). This range accommodates a comprehensive spectrum of sentiments. The BMSLSS has exhibited strong reliability and validity across numerous studies [47,48], affirming its credibility as an effective measurement tool. The internal consistency of the scale is satisfactory, with Cronbach’s alpha coefficient of α = 0.82 and McDonald’s omega coefficient of ω = 0.82.

#### 2.2.4. Stressful Life Events

The Life Events Checklist (LEC) [49] scale utilized in this study is a 28-item instrument designed to assess both controllable and uncontrollable stressful life events. Participants were asked to indicate whether they experienced any of these stressors in the two years following the onset of the COVID-19 pandemic in the United States in March 2020. For this study, 17 specific events were selected from the scale for assessment. Notable examples of these events include “divorce of parents” and “loss of a parent’s job” [50]. Previous research supports the reliability and validity of the LEC. For example, its two-week test–retest reliability was estimated at 0.72 for negative life events [51]. The convergent validity of the scale is good, with significant correlations to other established measures of stressful life events, including the Stressful Life Events Schedule for Children and Adolescents and the Life Events and Difficulties Schedule [52]. Further evidence of validity comes from significant associations between LEC scores and measures of youth outcomes for depression, conduct disorder, and oppositional defiant disorder [53,54].

The Life Events Checklist has been widely employed in studies investigating the impact of stressful life events on adolescents, particularly during the COVID-19 pandemic, to assess mental health outcomes such as anxiety, depression, and traumatic life events [55,56,57]. Although the internal consistency of the scale in this study was slightly lower (α = 0.68, ω = 0.65), its use remains appropriate given the scale’s broad coverage of diverse stressful events and the typical reliability coefficients for scales of this nature (The Life Events Checklist’s lower reliability coefficients, while below the conventional threshold of 0.70, are justified by its design to measure a wide range of stressful events, which naturally leads to lower internal consistency [58]. The dichotomous nature of the scale, focusing on frequency counts, also contributes to the lower reliability, which is common for such scales [59]. Furthermore, the scale’s extensive use during the COVID-19 pandemic supports its relevance and applicability to current research contexts [55,56,57]).

#### 2.2.5. Data Analysis

**Figure 1:**
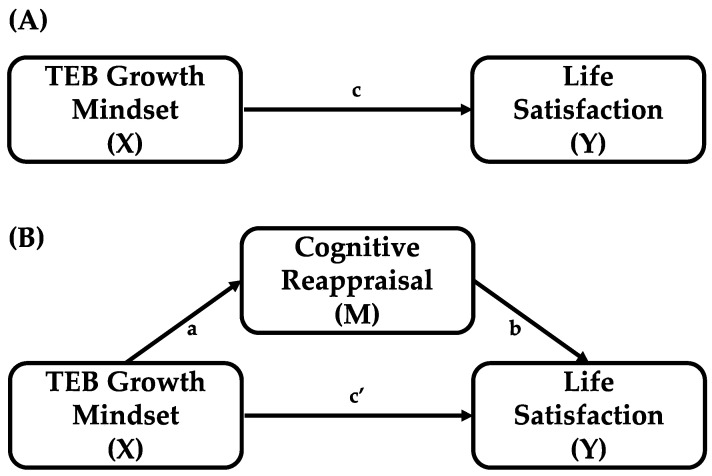
(**A**) Illustration total effect: X affects Y. (**B**) Illustration of the proposed mediation model: X affects Y indirectly through M. a-path represents the effect of mental health growth mindset on cognitive reappraisal. b-path represents the effect of cognitive reappraisal on life satisfaction. c-path and c’-path indicate the direct effect of mental health growth mindset on life satisfaction.

**Figure 2:**
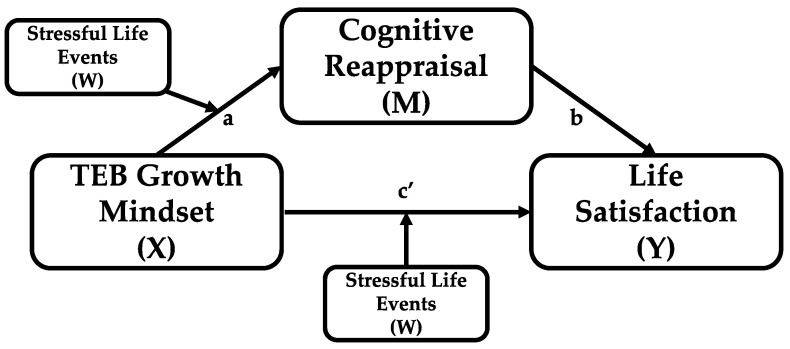
The figure depicts the following paths and effects: a-path (Moderated by W) represents the effect of mental health growth mindset on cognitive reappraisal, moderated by stressful life events. b-path represents the effect of cognitive reappraisal on life satisfaction. c’-path (Moderated by W) indicates the direct effect of mental health growth mindset on life satisfaction, moderated by stressful life events. Additionally, the conditional indirect effect illustrates the indirect effect of mental health growth mindset on life satisfaction through cognitive reappraisal at different levels of stressful life events.

Statistical analyses, including descriptive statistics and correlations, were conducted in SPSS v29. A *p* value < 0.05 was considered statistically significant. Mediation and moderated mediation were examined using the PROCESS macro for SPSS [41]. For mediation, Model 4 was specified with implicit theories of thoughts, emotions, and behavior (ITEB) as the predictor, cognitive reappraisal as the mediator, and life satisfaction as the outcome (see Figure 1 for the conceptual diagram). To test moderation, Model 8 was specified with stressful life events entered as a moderator (W). In this moderated mediation model, significant interactions were probed by estimating conditional direct and indirect effects of ITEB at values of stressful life events corresponding to the 16th, 50th, and 84th percentiles, representing low, moderate, and high stress in this sample (see Figure 2 for the conceptual diagram). PROCESS employs ordinary least squares regression and nonparametric bootstrapping; in this study, 5000 bootstrapped samples were used to generate bias-corrected 95% confidence intervals. An effect was considered statistically significant if the CI did not include zero [60,61].

In addition to the primary analyses, exploratory follow-up models were conducted to evaluate whether the mediation process was consistent across key demographic groups. PROCESS Model 59 was used to test whether gender or educational grade moderated the relations among ITEB, cognitive reappraisal, and life satisfaction. This model allowed us to examine whether the strength of the indirect association differed for boys versus girls or across grade levels, providing an assessment of the robustness of the mediation model within demographic subgroups. Descriptive statistics for ITEB, cognitive reappraisal, and life satisfaction were also calculated separately by gender and educational grade to provide additional context for group-level patterns.

## 3. Results

### 3.1. Descriptives and Correlations

The associations among implicit theories of thoughts, emotions, and behavior (ITEB; higher scores = stronger growth mindset), cognitive reappraisal, stressful life events, and life satisfaction were investigated using Pearson’s correlation analysis (two-tailed; Table 2). In this sample, a significant positive association between ITEB and cognitive reappraisal (*r* = 0.44, *p* < 0.001) and between ITEB and life satisfaction (*r* = 0.43, *p* < 0.001) indicating that adolescents who more strongly endorsed growth-oriented beliefs reported greater use of reappraisal strategies and higher life satisfaction. Life satisfaction was also found to be positively associated with cognitive reappraisal (*r* = 0.35, *p* < 0.001) and negatively associated with stressful life events (*r* = −0.26, *p* < 0.001). There were no significant associations between stressful life events and ITEB or cognitive reappraisal.

Descriptive statistics computed separately by gender and by educational grade indicated only small group differences across these variables (see Table 3), supporting the use of the full sample for the primary mediation analyses.

### 3.2. Mediation Model

We tested whether cognitive reappraisal mediated the association between ITEB and life satisfaction using PROCESS Model 4. The total effect of ITEB on life satisfaction was significant, *B* = 0.57, *SE* = 0.05, *t* = 11.70, *p* < 0.001, 95% CI [0.47, 0.67]. When cognitive reappraisal was added to the model, the direct effect of ITEB on life satisfaction remained significant, *B* = 0.45, *SE* = 0.05, *t* = 8.52, *p* < 0.001, 95% CI [0.35, 0.56], indicating partial mediation. Cognitive reappraisal was also positively associated with life satisfaction, *B* = 0.34, *SE* = 0.07, *t* = 5.15, *p* < 0.001, 95% CI [0.21, 0.48]. The indirect effect of ITEB on life satisfaction through cognitive reappraisal was significant, *B* = 0.12, Boot*SE* = 0.03, 95% CI [0.07, 0.18], as the confidence interval did not include zero. These findings suggest that adolescents who more strongly endorsed growth mindset beliefs about TEB reported higher life satisfaction, in part through their greater use of cognitive reappraisal strategies. As shown by the standardized coefficients in Table 4, the magnitudes of these paths fall within the small-to-moderate range commonly observed in prior adolescent research on implicit beliefs and emotion regulation. In particular, the effect of ITEB on reappraisal and the size of the indirect effect are comparable to those reported in studies linking emotion-related beliefs to regulatory processes and downstream adjustment [23,27,28]. Full regression results are presented in Table 4.

### 3.3. Moderated Mediation Model

Next, PROCESS Model 8 was used to test whether the indirect effect of ITEB on life satisfaction through cognitive reappraisal was moderated by stressful life events (see Figure 2 for the conceptual diagram). The interaction between ITEB and stressful life events on cognitive reappraisal (a-path) was not significant, *B* = 0.004, *SE* = 0.01, *p* = 0.68, 95% CI [−0.01, 0.02], indicating that stressful life events did not moderate the association between ITEB and cognitive reappraisal.

**Figure 3:**
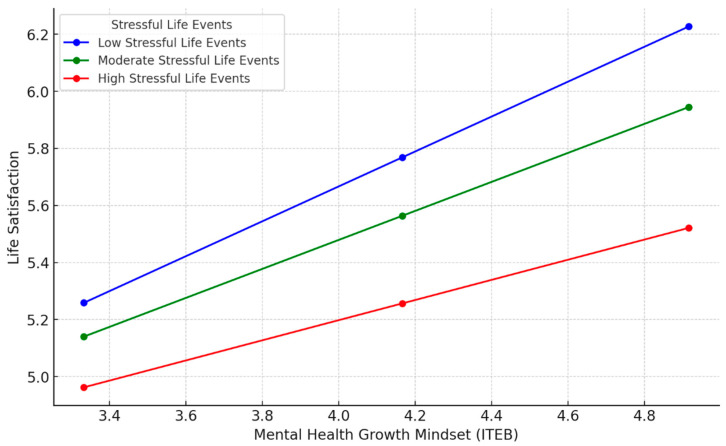
Conditional Effect of Stressful Life Events on the Relation Between ITEB and Life Satisfaction (c’-path).

However, the interaction between ITEB and stressful life events on life satisfaction (c’-path) was significant, *B* = −0.04, *SE* = 0.02, *p* = 0.007, 95% CI [−0.07, −0.01]. Conditional effects analyses revealed that the positive relation between ITEB and life satisfaction weakened as stressful life events increased: at low stress (16th percentile, 2.0), *B* = 0.54, *SE* = 0.06, *p* < 0.001, 95% CI [0.41, 0.67]; at moderate stress (50th percentile, 4.0), *B* = 0.46, *SE* = 0.05, *p* < 0.001, 95% CI [0.35, 0.56]; and at high stress (84th percentile, 7.0), *B* = 0.33, *SE* = 0.06, *p* < 0.001, 95% CI [0.21, 0.46]. Figure 3 illustrates this interaction.

The index of moderated mediation was not significant, *B* = 0.001, Boot*SE* = 0.004, 95% CI [−0.01, 0.01], indicating that the indirect effect of ITEB on life satisfaction via cognitive reappraisal was consistent across stress levels. In sum, cognitive reappraisal partially mediated the association between ITEB and life satisfaction, and the direct association was moderated by stressful life events, such that the positive relation was attenuated at higher levels of stress. No moderation was observed on the a-path.

### 3.4. Exploratory Moderation Analyses by Gender and Educational Grade

Exploratory moderation analyses were conducted to evaluate whether the mediation model varied across demographic subgroups. Neither gender nor educational grade moderated the association between ITEB and cognitive reappraisal (all *p*s > 0.43) or the association between cognitive reappraisal and life satisfaction (all *p*s > 0.62). The direct effect of ITEB on life satisfaction also did not differ by gender or grade (*p*s > 0.34).

Conditional indirect effects were significant across all groups and were similar in magnitude, indicating that the indirect pathway from ITEB to life satisfaction through cognitive reappraisal was stable for girls and boys and across grade levels. Together, these findings suggest that the mediation process operated consistently across demographic subgroups in this sample.

## 4. Discussion

This study examined whether cognitive reappraisal mediates the association between ITEB and adolescent life satisfaction, and whether stressful life events moderate these pathways. Results showed that reappraisal partially accounted for the ITEB-life satisfaction link. Though stress did not moderate the indirect path, it weakened the direct association between ITEB and life satisfaction, suggesting that the benefits of growth-oriented beliefs are less reliable under high stress.

These findings support theoretical accounts positioning mindsets as motivational systems that guide self-regulation. The SOMA model [12] proposes that incremental beliefs influence operating processes, including the strategies deployed under strain. Consistent with this model, adolescents who endorsed more growth-oriented ITEB reported greater use of reappraisal, which in turn predicted higher life satisfaction. Prior work on implicit theories of emotion shows a parallel pattern, with growth beliefs linked to greater reappraisal and lower depressive symptoms [27,28]. The present study extends this evidence by showing that beliefs about the malleability of internal states broadly—not just emotions—relate to well-being through adaptive regulatory tendencies.

Importantly, the findings also align with contemporary resilience frameworks emphasizing that youth well-being reflects the joint operation of assets and stressors, rather than the absence of pathology alone. Dual-factor models highlight that mental health is best represented by two related but distinct continua—subjective well-being and psychological distress [62,63]. Within this framework, ITEB may operate as both a promotive factor that supports well-being and as a potential vulnerability factor when contextual affordances are limited. Our results illustrate this duality: under lower stress, adolescents with stronger growth beliefs reported higher life satisfaction, yet under higher stress, the direct benefits of these beliefs diminished. This pattern is consistent with resilience science, which argues that adaptive systems—including close relationships and stable learning environments—must remain intact for individual assets such as self-regulation to function effectively [4]. When stress is high and external supports are strained, growth beliefs may be harder to enact, reducing their observable benefit.

This interpretation resonates with the mindset × context perspective, which proposes that beliefs exert their strongest influence when environments provide opportunities to act on them [15]. Stressful contexts may restrict these affordances, thereby weakening the direct link between incremental beliefs and well-being. Yet, consistent with resilience portfolio models [64], growth-oriented beliefs may still buffer specific interpersonal stressors, even when their global influence is constrained. Although our stress measure was broad, research on domain-specific stress (e.g., peer stress) has shown that ITEB can protect life satisfaction under socially threatening conditions [65]. Together, these findings suggest that ITEB may function as a protective factor in contexts where adolescents can exert some agency but may offer less benefit in circumstances characterized by chronic, uncontrollable stress.

The absence of moderation on the indirect path also warrants attention. One explanation lies in measurement: our stress index captured event counts rather than subjective appraisals of severity or controllability, which are more proximal to coping responses [5,29]. Trait measures of reappraisal such as the ERQ-CA may further obscure situational variation compared to state-based assessments [36]. Low correlations between stressful life events and both ITEB and reappraisal in this sample also reduced the likelihood of detecting moderation. A further methodological consideration involves the internal consistency of the Life Events Checklist, which was modest in the present sample (α = 0.68, ω = 0.65). Although lower reliability is common for event-checklist measures that assess diverse, infrequent, and heterogeneous stressors, this level of measurement error likely attenuated associations involving stress and reduced statistical power for detecting interaction effects. This limitation is especially important for moderated mediation models, where unreliability in the moderator can substantially weaken both the a-path interaction and the index of moderated mediation. Thus, the absence of moderation on the indirect pathway should be interpreted cautiously, as true effects may have been obscured by measurement constraints. Future studies would benefit from stress measures with stronger psychometric properties, particularly instruments assessing subjective perceptions of stress severity, controllability, or chronic strain, which are theoretically more proximal to regulation and less susceptible to attenuation. Multi-method or repeated assessments, including ecological momentary sampling, may further enhance sensitivity to the dynamic interactions among beliefs, stress, and real-time strategy use.

These results carry practical implications for youth mental health promotion. Interventions that integrate growth-mindset messaging with explicit reappraisal training may be especially impactful. Brief single-session interventions teaching that thoughts, feelings, and behaviors can change reduce depressive symptoms [23], while reappraisal training enhances adaptive regulation [45]. Embedding these elements within school-based social-emotional learning (SEL) and prevention programs may help support both components of dual-factor mental health—reducing symptoms and strengthening well-being. At the same time, because high stress appears to blunt the broader benefits of incremental beliefs, universal SEL programming should be complemented with targeted supports for highly stressed adolescents. In line with resilience-informed recommendations, these supports might include developmentally attuned adult relationships, opportunities for relational connection, and adjustments to school and home environments that reduce chronic strain and bolster everyday coping resources [4]. Such efforts may help restore the adaptive systems needed for growth beliefs to translate into well-being.

These implications may be especially relevant for adolescents, for whom both mindset formation and regulatory skills are undergoing rapid developmental change. Early-to-mid adolescence is marked by heightened emotional reactivity and increased sensitivity to social evaluation [66], alongside ongoing maturation of prefrontal regulatory systems that support deliberate control over thoughts and emotions [67]. Stress exposure also tends to increase during this period as academic demands intensify and peer contexts become more complex [68]. Thus, understanding how growth-oriented mental health beliefs intersect with adolescents’ developing capacity for cognitive reappraisal provides insight into mechanisms that may support well-being during a sensitive window of neurobiological and social transition.

Several caveats should be noted when drawing conclusions. First, the cross-sectional design prevents determination of directionality from the predictor to the outcome. We were also not able to test bidirectional influences between variables in the model, which is particularly important during developmental transitions when beliefs and regulation are most malleable. Longitudinal designs will be necessary for establishing antecedent–consequence links or reciprocal pathways. Second, reliance on self-report raises concerns about shared method variance and bias. Multi-informant approaches—including parent, teacher, and observational reports—would provide a more comprehensive account of how beliefs translate into coping in daily life. In addition, exclusive reliance on single-reporter questionnaires increases susceptibility to common method bias and self-report inflation, particularly for constructs that tap internal states or socially desirable regulatory tendencies. Future studies incorporating cross-informant data and performance-based or observational measures of regulation would help mitigate these concerns. Third, while the pandemic context offered a natural test of coping under pressure, its unique demands may limit generalizability. The pandemic introduced atypical stress exposures—such as social isolation, rapid routine disruption, and health-related uncertainty—that may have amplified or constrained adolescents’ opportunities to act on their beliefs. Replication in more typical stress contexts will therefore be essential for determining whether these patterns are specific to this historical moment or reflect broader developmental processes.

## 5. Conclusions

This study shows that adolescents who endorse growth-oriented beliefs about thoughts, emotions, and behavior report greater life satisfaction, in part because they more often use cognitive reappraisal. This mediational pathway was consistent across stress levels, suggesting that reappraisal is a robust mechanism linking mindset to well-being. At the same time, the direct benefits of incremental beliefs diminished under high stress, underscoring that beliefs alone are insufficient without supportive contexts. Given the cross-sectional design, the present findings reflect correlational patterns rather than directional effects; future longitudinal and experimental work is needed to clarify how these processes unfold over time. Taken together, these findings affirm the dual importance of fostering growth-oriented mindsets and concrete regulation strategies, while also highlighting the need to consider contextual affordances. By integrating motivational beliefs, coping processes, and stress exposure, this work offers actionable guidance for strengthening resilience and supporting adolescent well-being.

## Figures and Tables

**Table 1:** Demographic Information.

Variable	Category	n (%)
Grade Level	9th Grade	74 (12.0%)
	10th Grade	115 (18.7%)
	11th Grade	147 (23.9%)
	12th Grade	284 (45.4%)
School Setting	Suburban	319 (51.5%)
	Urban	205 (33%)
	Rural	96 (15.5%)
Gender	Female	307 (49.5%)
	Male	270 (43.5%)
	Gender-Nonconforming/Variant	36 (5.8%)
	Not Disclosed	7 (1.1%)
Living Arrangements	Both Parents	406 (65.5%)
	Mother or Mother Figure	162 (26.1%)
	Father or Father Figure	23 (3.7%)
	Other Adults	29 (4.7%)
Racial Identity	White	256 (41.3%)
	Black	120 (19.4%)
	Hispanic/Latinx	119 (19.2%)
	Multiple Selections	56 (9.0%)
	Asian	40 (6.5%)
	Biracial or Multiracial	22 (3.5%)
	American Indian/Alaska Native	6 (1.0%)
	Native Hawaiian or Other Pacific Islander	1 (0.2%)
Geographic Region	South	223 (36.0%)
	West	167 (27.0%)
	Midwest	118 (19.0%)
	Northeast	112 (18.0%)

Note. The sample size (n) for this study was 620 participants. The table includes distributions across grade levels, school settings, gender, living arrangements, racial identity, and geographic regions.

**Table 2:** Descriptive Statistics and Bivariate Two-Tailed Pearson Correlations Between Key Variables.

Variable	Mean (SD)	*α*	ω	2	3	4
1. ITEB	4.10 (0.84)	0.89	0.89	0.44 **	−0.07	0.43 **
2. Cognitive reappraisal	3.38 (0.67)	0.78	0.77	-	−0.03	0.35 **
3. Stressful life events	4.59 (2.85)	0.68	0.65	-	-	−0.26 **
4. Life satisfaction	5.48 (1.25)	0.82	0.82	-	-	-

Note. Implicit theories of thoughts, emotion, and behavior = ITEB. ** *p* < 0.01.

**Table 3:** Descriptive Statistics for Primary Study Variables by Gender and Grade.

Group	n	ITEB M (SD)	Cognitive Reappraisal M (SD)	Life Satisfaction M (SD)
**Gender**				
Girls (1)	307	4.06 (0.83)	3.33 (0.65)	4.23 (1.06)
Boys (2)	270	4.20 (0.79)	3.47 (0.67)	4.62 (1.16)
**Grade**				
9th grade	74	4.01 (0.94)	3.30 (0.66)	4.47 (1.24)
10th grade	115	4.01 (0.83)	3.32 (0.67)	4.36 (1.07)
11th grade	147	4.09 (0.85)	3.39 (0.68)	4.28 (1.23)
12th grade	284	4.16 (0.81)	3.41 (0.66)	4.43 (1.05)

**Table 4:** Results of the mediation analysis.

	*B*	*SE*	β	*t*	*p*	CI
Path a: ITEB → Cognitive Reappraisal	0.35	0.44	0.03	12.25	<0.001	[0.29, 0.41]
Path b: Cognitive Reappraisal → Life Satisfaction	0.34	0.07	0.20	5.15	<0.001	[0.21, 0.48]
Total Effect (path c): ITEB → Life Satisfaction	0.57	0.05	0.43	11.70	<0.001	[0.47, 0.67]
Direct Effect (path c’): ITEB → Life Satisfaction	0.45	0.05	0.34	8.52	<0.001	[0.35, 0.56]
Indirect Effect: ITEB → Cognitive Reappraisal → Life Satisfaction	0.12	0.03	0.09	-	-	[0.07, 0.18]

***Note.****B* = unstandardized coefficients; β = standardized coefficients. All variables were standardized prior to estimation of β values. Bootstrap sample size = 5000. PROCESS uses ordinary least squares regression; therefore, global SEM model-fit indices (e.g., CFI, RMSEA) do not apply.

## Data Availability

The raw data supporting the conclusions of this article will be made available by the authors on request.
